# Supplementary material for: New insights into the spatial organization, stratigraphy and human occupations of the Aceramic Neolithic at Ganj Dareh, Iran
Source: PLoS One. 2021 Aug 18;16(8):e0251318. doi: 10.1371/journal.pone.0251318 (PMC8372917; doi:10.1371/journal.pone.0251318)
Supplement: S1 File — (ZIP) [file pone.0251318.s001.zip › lithic_clay.pdf]

Lithic_clay

01/06/2020

Preparing the workspace Packages required for the script

```
library(ggplot2)
library(scales)
library(ggpmisc)
```

Setting the work directory and importing/checking data

```
setwd("C:/Users/simon/Desktop/lithic_clay/")
data <- read.csv2("GD_lithic_clay.csv", header = TRUE, sep = ";", dec = ",")

head(data)
```

```
##   Level n_retouched_lithics n_cores tot_n_lithics Lithic_volumetric_density
## 1  A-01                  86      14          1316                     80.64
## 2  B-01                  69      13          1211                    102.28
## 3  B-02                  31      12           688                    119.44
## 4  C-01                  55       8           817                     88.04
## 5  C-02                  98      23          1311                    130.06
## 6  D-01                  19       2           166                    171.13
##   percentage_retouch n_zoomorphic_fig n_anthropo_fig n_geom_fig_tokens
## 1               6.53                2              0                13
## 2               5.70                1              0                 0
## 3               4.51                0              0                 1
## 4               6.73                1              1                 3
## 5               7.48                1              1                 5
## 6              11.45                0              0                 0
##   n_non_geom_fig tot_n_figurines
## 1              7              22
## 2              1               2
## 3              1               2
## 4              1               6
## 5              1               8
## 6              1               1
```

Removing layers with no lithics remains

```
lithics <- subset(data, Lithic_volumetric_density > 0)
```

Creating the scatterplot Creating the scatterplot with trendline for the lithic volumetric density and the retouch frequency. In the same step, using ggplot2 to measure and display the r squared value and the p-value of the trendline.

```
plot1 <- ggplot(lithics, aes(x= Lithic_volumetric_density, y= percentage_retouch, label= Level)) +
  geom_point(colour = "dodgerblue3", size = 2.5) +
  geom_text(aes(label=Level), hjust=-0.1, vjust=1.1, size = 3) +
  labs(x = "Lithic volumetric density", y = "Retouch frequency") +
  scale_x_continuous(trans = "log10", limits = c(1, 1000), breaks = c(1, 10, 100, 1000)) +
  scale_y_continuous(trans = "log10", limits = c(1, 100), breaks = c(1, 10, 100)) +
  theme_bw() +
  theme(panel.grid.major = element_line(colour = "#d3d3d3"),
        panel.grid.minor = element_blank(),
        axis.text.x = element_text(colour = "black"),
        axis.text.y = element_text(colour = "black")) +
  stat_smooth(method = "lm", formula = y ~ log(x), colour = "dodgerblue4",
              size = 1, fill = "gray82", linetype = "dashed") +
  stat_poly_eq(aes(label = paste(..rr.label..)), #measure and display the R2
               label.x = 0.09, label.y = 0.17,
               formula = y ~ log(x), parse = TRUE, size = 4) +
  stat_fit_glance(method = 'lm', #measure and display the p-value
                  method.args = list(formula = y ~ log(x)),
                  geom = 'text', aes(label = paste("P-value = ", signif(..p.value.., digits = 4), sep =
                  label.x = 0.31, label.y = 0.21, size = 4)
```

Display the scatterplot

```
plot(plot1)
```

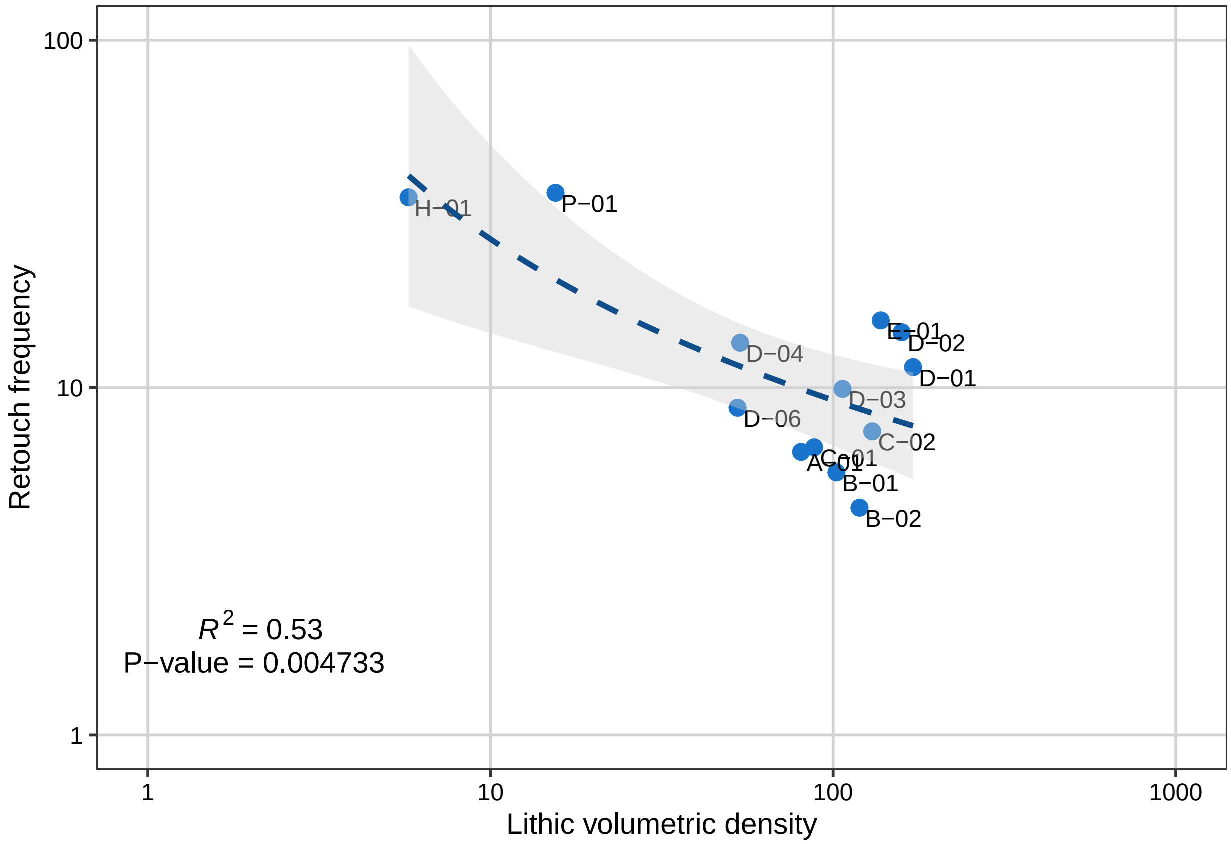

Correlation between the lithics and ceramics Create a linear model for the total number of lithics and the total number of ceramics and display the p-value and the r-squared value.

```
model <- lm(tot_n_lithics ~ tot_n_figurines, data = data)
summary(model)
```

```
##
## Call:
## lm(formula = tot_n_lithics ~ tot_n_figurines, data = data)
##
## Residuals:
##    Min     1Q Median     3Q    Max
## -570.8 -310.4 -109.6  270.9  689.7
##
## Coefficients:
##                 Estimate Std. Error t value Pr(>|t|)
## (Intercept)       422.40     142.68   2.960   0.0119 *
## tot_n_figurines    49.46      19.92   2.482   0.0288 *
## ---
## Signif. codes:  0 '***' 0.001 '**' 0.01 '*' 0.05 '.' 0.1 ' ' 1
##
## Residual standard error: 419.5 on 12 degrees of freedom
## Multiple R-squared:  0.3393, Adjusted R-squared:  0.2842
## F-statistic: 6.163 on 1 and 12 DF,  p-value: 0.02883
```
