# Supplementary figures and images for: New insights into the spatial organization, stratigraphy and human occupations of the Aceramic Neolithic at Ganj Dareh, Iran
Source: PLoS One. 2021 Aug 18;16(8):e0251318. doi: 10.1371/journal.pone.0251318 (PMC8372917; doi:10.1371/journal.pone.0251318)

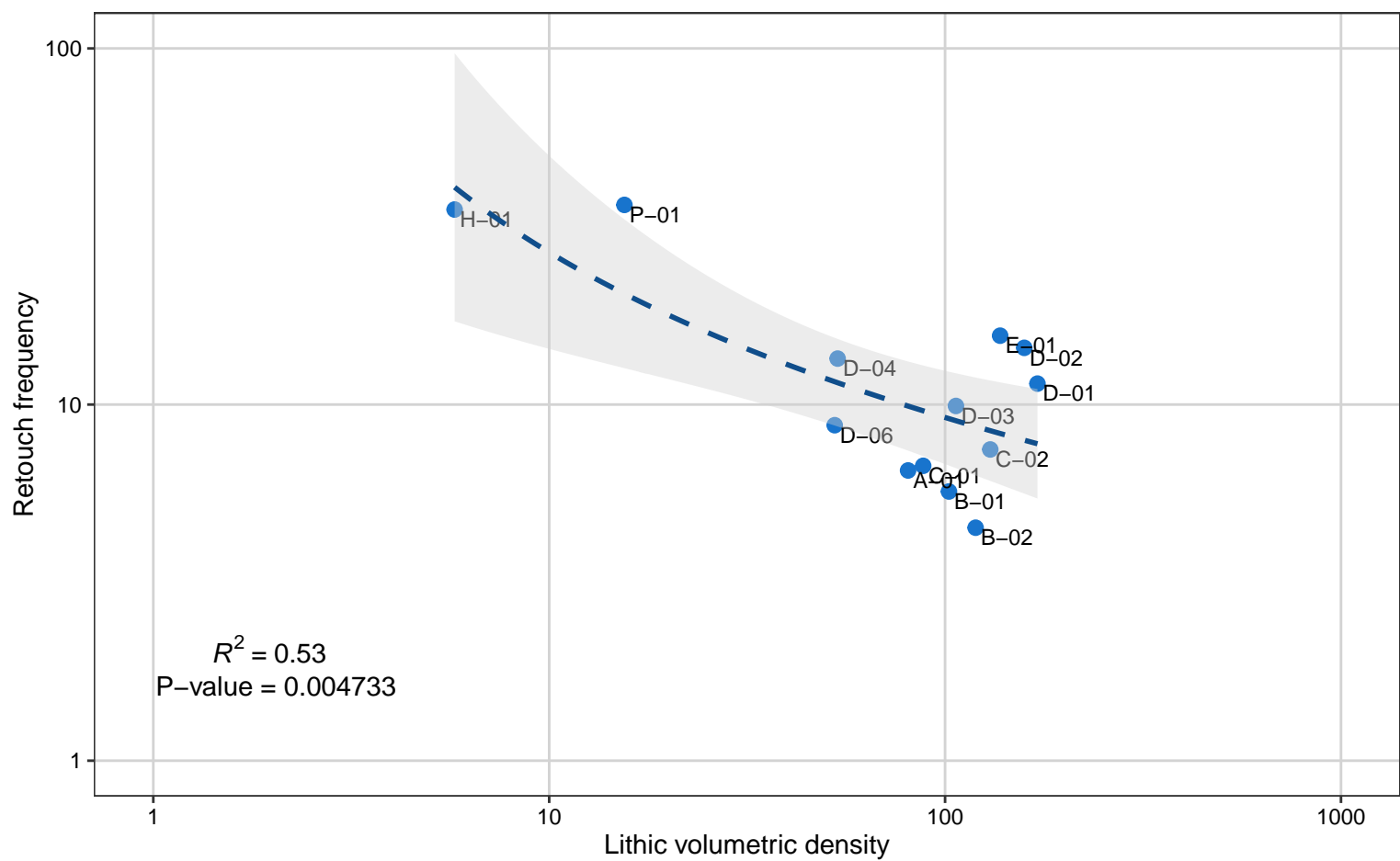

Supplement: S1 File — (ZIP) [file pone.0251318.s001.zip › plot.pdf]
