# Supplementary material for: New insights into the spatial organization, stratigraphy and human occupations of the Aceramic Neolithic at Ganj Dareh, Iran
Source: PLoS One. 2021 Aug 18;16(8):e0251318. doi: 10.1371/journal.pone.0251318 (PMC8372917; doi:10.1371/journal.pone.0251318)
Supplement: Attachment — Submitted filename: Review of Riel-Salvatore et al..docx [file pone.0251318.s003.docx]

Review of Riel-Salvatore

This initial report on the analysis of the newly discovered excavation reports from the famous site of Ganj Dareh is a very welcome addition to the growing number of studies of the Neolithic in Central Zagros. The Central Zagros was once held to be a major center for initial domestication and agricultural emergence in the Near East. With the interruption of archaeological research in the region in the late 1970s the focus of research shifted to the Levant and the Central Zagros, and indeed the entire eastern half of the Fertile Crescent region, was portrayed as a backwater, bit-player in the story of the Near Eastern Neolithic. Thanks to a combination of new investigations in the region and the reanalysis of material from earlier excavations (like this one), we are seeing a major rehabilitation of the Central Zagros as a major player in the multi-centric process of domestication and agricultural emergence that took place across the entire arc of the Fertile Crescent during the Early Holocene.

Ganj Dareh has long been recognized as an important site for understanding the Iranian Neolithic. Yet other than the analysis of the faunal remains by Hesse and then Zeder, there has been almost no detailed published account of the stratigraphy and material culture recovered in early excavations at this site. This careful analysis of the excavation reports recently discovered at the University of Montreal is then a significant contribution to our growing understanding of the Iranian Neolithic and its role in the emergence of agricultural economies and village life in the Near East. This report fills in major gaps in our understanding of Ganj Dareh and its place in the process of Neolithization in the Near East by providing at long last a report on the stratigraphy of this complicated tell site, as well as information on the lithics, clay objects, chronology, and burials gleaned from these records. Moreover, the discovery of the two previously unrecognized lower levels and their possible affiliation with a preceding Epipaleolithic period that may predate the upper architectural levels of the site may at long last resolve a long open question about an apparently anomalous early date from basal levels and, more importantly, help brings into sharper focus the picture of the foundational Early Holocene adaptations in the Zagros that in all likelihood saw the initial steps toward both plant and animal domestication in this region. As such, I fully support publication of this report in PLOS ONE.

I do, however, have a few suggestions for revision.

My major recommendation is that more be done to set this study within the broader context of the Neolithic in the Central Zagros. The picture of agricultural emergence in the region and Ganj Dareh’s place within it presented here seems somewhat dated and does not make effective use of recent work in the region. In their discussion of the archaeobotanical remains from the site, for example, the authors downplay the role of domesticated crops at Ganj Dareh maintaining that the only evidence of food production at the site is the presence of domesticated goats. To support this thesis they cite van Zeist’s hypothesis that the domestic barley that increases in prevalence throughout the occupation of the site was likely not for human consumption but used as animal fodder. And yet there is now increasing evidence for the active engagement of people in the region in low level food production that involves native grasses and domestic barely documented at Chogha Golan and other recently excavated sites in the region. And while the barley recovered from dung burned as fuel at Ganj Dareh (and other contemporary sites) was likely to have been fed to goats, that doesn’t mean that humans didn’t consume barley as well.

They claim that the lentils recovered from the site were likely wild collected resources on the basis of their small size. This ignores important work by Weiss in the Levant that makes the case for early cultivation/domestication of lentils despite their small size research that indicates that an increase in seed size is a lagging indicator of domestication in this species. They also fail to cite important work by Savard and others that shows that people in this region had a heavy focus on pulses (including lentils) reaching back into the Epipaleolithic indicative of the management if not indigenous domestication of these plants.

Moreover, while the faunal remains from Ganj Dareh do indeed show the earliest clear evidence of goat management in the harvest profiles congruent with strategies of modern day herders, it is unlikely that the site captures the initial phases of goat domestication as implied here. The fact that this harvest strategy is fully in-place in the earliest levels at the site suggest that this practice considerably pre-dates the architectural levels at the site. This possibility is reinforced by data from southeastern Anatolia that indicates the presence of managed goats by 10,500 cal. BP and for the importation of clearly managed goats to Cyprus around the same time.

The discussion of the environmental setting of the site fails to make reference to recent paleo-environmental reconstructions by Roberts and Asouti that have considerably revised that of Van Zeist in the cited 1984 publication.

Similarly, more discussion is needed of the importance of the exciting discovery of the previously unrecognized basal levels of the site. The identification of these levels (along with the intriguing lithic evidence for greater mobility than in the later levels E though A) fits well into the emerging picture of the Epipaleolithic in the region documented by more recent excavations at Asiab, basal Sheikh-e-Abad, Chia Sabz, and Chogha Golan, as well as by older excavations at Abdul Hosein. At all these sites we find evidence for an early phase of the settlement history of the region in which foragers made use of the increasing abundance of resources that accompanied Early Holocene climate amelioration. These discoveries suggest that the Central Zagros is likely part of a broader cultural phenomenon spread across the Eastern Fertile Crescent region – Peasnall’s round house phase – evidenced at contemporary sites such as Zawi Chemi, Karim Shahir, Hallan Çemi and even basal levels at Çayönü. These are the sites (and the time period) that likely served as the starting point for the food production strategies that are firmly in place by the time of the establishment of the architectural levels at Ganj Dareh.

The picture that emerges, then, is that these newly discovered levels at the site represent this initial phase of Early Holocene settlement history in the region, while the architectural levels represent a later phase in which low level food production that feature a mix of domestic, managed, and wild resources is well established among a network of fully sedentary villages spread across the Central Zagros. More discussion of this broader context would help underscore the importance of this analysis of these archival materials in adding to the growing understanding of the Neolithic in the Central Zagros.

On a less substantive level, it would be good to have a clearer statement whether this paper represents just an initial phase of the study of the materials discovered in Montreal – that of the excavation records and not of the artifacts that accompany these records. Are these all the field excavation reports, plans, photos, etc. or just a selection of them? It would also be good to have an account of what the archival materials consist of as well as a discussion of any plans for future study of these archives. Some idea of the size of the artifact collections in Canada and plans for future work on them would also be helpful.

The paper would also benefit from more illustrations of the lithics (especially the purported Paleolithic stone tools) and the clay objects discussed in the text.
