# Supplementary material for: New insights into the spatial organization, stratigraphy and human occupations of the Aceramic Neolithic at Ganj Dareh, Iran
Source: PLoS One. 2021 Aug 18;16(8):e0251318. doi: 10.1371/journal.pone.0251318 (PMC8372917; doi:10.1371/journal.pone.0251318)
Supplement: Attachment — Submitted filename: Response to reviewers_Apr10.docx [file pone.0251318.s004.docx]

Monday April 10, 2021

Montréal, QC

Dear Professor Biehl,

We write the following rebuttal letter to respond to the comments on our manuscript “New Insights into the Pre-Pottery Neolithic Spatial Organization, Stratigraphy and Human Occupations of Ganj Dareh, Iran” which you transmitted to us on Oct. 1, 2020, indicating that addressing them involved making minor revisions to the manuscript, and following the return of our revised version due to editorial issues on Apr. 7, 2021. We begin by thanking the reviewers for their extremely positive assessments and for their constructive feedback. We also would like to apologize for the time it took us to return the revised manuscript, which was in part due to two of the co-authors coming down with COVID-19 and tracking long-term symptoms since.

Prof. Özdoğan’s comments were minimal and straightforward to address. Concerning the animated 3D reconstruction of the site’s stratigraphy, we have added on p. 17 a comment about the detail this animation brings to our understanding of the site’s overall layout and changing occupation focus, which is more difficult to appreciate using static plans. On p. 47, we have also added a paragraph explaining the importance of conserving analog hard copies of field documentations, in accordance with best practices in legacy collection management. We also detail how this fits into a broader plan of future work for the Ganj Dareh archive at UdeM in an additional two concluding paragraphs on pp. 46-47 detailing the next steps of this work over the coming years, in response to Dr. Zeder’s comments. Lastly, concerning the issue of lithic density being influenced by area function, we have already cited on p.31 the relevant literature demonstrating the validity of this approach in a variety of archaeological contexts, but we have added a qualifying sentence that highlights that any lithic assemblage is liable to be biased thusly, whether it is analyzed using the WABI as we do here, or using more traditional techno-typological approaches.

Turning to Dr. Zeder’s very helpful comments and suggestions, we have updated the relevant sections of the text and incorporated her suggested literature on research on the Aceramic Neolithic of the Zagros and the Eastern Fertile Crescent. On pp. 7-8, we have updated the discussion about the likely importance of domesticated crops at Ganj Dareh and other nearby Aceramic Neolithic sites, focusing on pulses as well as recently published data on triticoid grains. This is complemented by a broader discussion of the context for goat domestication in Anatolia and neighboring region at that time to better situate the evidence for this phenomenon at Ganj Dareh on p. 6. Additional context has also been added by reference to recent paleoenvironmental research on the Aceramic Neolithic of the Zagros on p. 3. In cultural terms, we have added a section on p. 6 that explains how Ganj Dareh was the full expression of a cultural phenomenon that unfolded across the Eastern Fertile Crescent in the preceding centuries. We have, in particular, expanded the discussion on pp. 33, about how the pattern of possible Epipaleolithic assemblages at the base of Ganj Dareh corresponds with has been documented at sites in the region.

As concerns the illustrations of the lithics and clay objects, these are part of ongoing unpublished efforts (MSc and PhD theses) and we prefer to reserve them for publication where the students who produced them can be first author, especially since they are simply the subject of basic quantification here, not detailed analysis. We felt comfortable with this decision since Prof. Özdoğan did not mention this as an essential revision. We have also taken the liberty to add a few references to new studies pertaining to the archaeology of Ganj Dareh and the Central Zagros that have been published since we submitted our manuscript this past summer and have made an editorial choice to replace the label “Pre-Pottery Neolithic” used in our original submission by “Aceramic Neolithic” throughout.

Turning to your editorial comments and the technical issues concerning Figures 2a and 2b, we have modified Fig. 2a in accordance to your specification and removed its complement originally submitted as Fig. 2b. As such, we have relabeled that revised figure Fig. 2 (and modified referring text on p. 16 and edited the legend for it on p. 59). Fig. 2 in its current form does not contain any copyrighted imagery, satellite or otherwise. We have also modified the acknowledgments on p. 48 to remove mentions to funding sources and to thank the reviewers and two other colleagues who have provided help in contextualizing the site. On p. 11, we have added a sentence indicating we are studying the Ganj Dareh material with the permission of the Faculty of Arts and Sciences at Université de Montréal. Finally, we have removed any references to other funding sources except the CFI grant to Riel-Salvatore.

All of the modified text has been highlighted in red, with removals highlighted in red and double-crossed through. All of the numbering of the references has been changed in order to account for the addition of over 25 new sources.

We hope you will find what have addressed your and the reviewers’ comments satisfactorily and look forward to hearing about your final decision about publishing our manuscript in PLOS ONE in the near future.

On behalf of my co-authors and I,

Bien cordialement,

Julien Riel-Salvatore, PhD

Professeur titulaire
